# Low Expression of IL-10 in Circulating Bregs and Inverted IL-10/TNF-α Ratio in Tears of Patients with Perennial Allergic Conjunctivitis: A Preliminary Study

**DOI:** 10.3390/ijms20051035

**Published:** 2019-02-27

**Authors:** Alberto Salazar, Israel Casanova-Méndez, Michele Pacheco-Quito, Henry Velázquez-Soto, Julio Ayala-Balboa, Enrique O. Graue-Hernández, Jeanet Serafín-López, María C. Jiménez-Martínez

**Affiliations:** 1 Departamento de Inmunología, ENCB, Instituto Politécnico Nacional, 11340 Ciudad de México, Mexico; alberto.salazar@institutodeoftalmologia.org (A.S.); jeaserafin@hotmail.com (J.S.-L.); 2 Department of Immunology and Research Unit, Institute of Ophthalmology “Conde de Valenciana Foundation”, 06800 Mexico City, Mexico; israel.casanova@elconde.org (I.C.-M.); henry.velazquez@institutodeoftalmologia.org (H.V.-S.); julbalboa@gmail.com (J.A.-B.); 3 Cornea and Refractive Surgery Department, Institute of Ophthalmology “Conde de Valenciana Foundation”, 06800 Mexico City, Mexico; michelepacheco86@hotmail.com (M.P.-Q.); egraueh@gmail.com (E.O.G.-H.); 4 Department of Biochemistry, Faculty of Medicine, National Autonomous University of Mexico. P.O. Box 70159, 04510 Mexico City, Mexico

**Keywords:** IL-10, TNF-α, tears, Bregs, allergic conjunctivitis

## Abstract

Allergic conjunctivitis (AC) is one of the most common ophthalmological disorders seen in clinical practice. Growing evidence from recent years suggests that a subset of IL-10-expressing B cells is involved in inflammatory allergic diseases. In this study, we aimed to evaluate the potential involvement of blood Bregs cells in perennial allergic conjunctivitis (PAC), and interleukins (IL)-1β, IL-6, IL-8, IL-10, and IL-12, and tumor necrosis factor (TNF)-α, were measured in tear samples and compared with healthy controls (HC) using flow cytometry. Non-significant differences in CD19^+^IL-10^+^ cell frequency between PAC patients and healthy controls (HC) were observed. Nevertheless, when we analyzed the mean fluorescence intensity (MFI) of IL-10 on CD19^+^CD38^Lo/Med/Hi^-gated cells, we observed a significant decrease in MFI in all Bregs subsets in PAC patients. Additionally, tear cytokines showed 2.8 times lower levels of IL-10 than TNF-α in PAC patients when compared to HC. Our findings demonstrate an immunological dysregulation in patients with allergic conjunctivitis, characterized by the low expression of IL-10 in circulating CD19^+^CD38^+^ Bregs subsets and an inverted tear IL-10/TNF-α ratio, promoting a local pro-inflammatory microenvironment. These findings highlight the novel pathologic changes involved in ocular allergic diseases. Understanding systemic and local mechanisms will aid the design of immunomodulating therapeutics at different levels.

## 1. Introduction

Allergic conjunctivitis (AC) is one of the most common ophthalmological conditions seen in clinical practice [1]. The prevalence varies from country to country, with rates between 15% and 40% [2], and children being the most affected population [3]. Clinically, it is characterized by bilateral injection of the conjunctiva with itching as the predominant symptom, and it is often also associated with nasal symptoms [4]. Ocular allergy may present as severe forms of chronic inflammation affecting the cornea and conjunctiva in atopic keratoconjunctivitis (AKC) and vernal keratoconjunctivitis (VKC); or mild forms of conjunctival inflammation, with periods of absence of damage in seasonal allergic conjunctivitis (SAC); or with mild persistent inflammation in perennial allergic conjunctivitis (PAC) as the acute form and the most frequent type of AC [1].

Damage to the conjunctiva appears to be mediated by the activation of CD4^+^ T cells by environmental allergens [2,5]. Circulating T-helper cells from patients with chronic forms express CD30 on their cell surface and produce large quantities of IL-4 and IL-5 compared with IFN-γ after allergen-specific stimulation, favoring a microenvironment related to Th2 over a Th1 response [6]. The Th1/Th2 microenvironment explains the injury mechanisms to the conjunctiva and cornea reported in those patients [1]. On the other hand, in acute forms, the cytokines IL-5, IL-6, and IL-8 are released after allergen-specific stimulation, favoring a microenvironment related to Th2 inflammation, which is characteristic in SAC and PAC [7,8].

Recently, we reported an imbalance between helper effector cells and Treg cells in PAC patients, with a higher frequency of Th2 cells in transit to the conjunctiva (CD4^+^CCR4^+^CCR9^+^) than circulating Treg (CD4^+^CD25^+^FoxP3^+^) cells [8]. The role of Tregs cells in the regulation of allergic diseases has been well recognized, and their lack of function or absence contributes to Th2-mediated inflammation [9,10]. In the last few years, growing evidence has suggested that the induction of Tregs is controlled by a subset of IL-10-expressing B cells (Bregs or B10 cells) [11,12,13]. Inhibiting the activation and expansion of pathogenic cells through IL-10 is the main function of Bregs; remarkably, a lack of Bregs cells is involved in autoimmunity [14,15], and a functional deficiency of IL-10^+^ B cells occurs in allergic airway inflammation [16]. This work aims to evaluate the potential involvement of IL-10^+^ B cells in allergic conjunctivitis and their impact on ocular changes.

## 2. Results

### 2.1. Frequency of CD19+IL-10+ B Cell Subsets in Peripheral Blood

We began by determining the percentage of CD19^+^IL-10^+^ B cells in the peripheral blood of 16 patients with PAC and 8 healthy controls (HC). Demographical characteristics of both groups are depicted in Table 1, and ophthalmological characteristics of patients and healthy controls are described in Tables S1 and S2. As expected, the percentage of CD19^+^ B cells was similar among patients with PAC and HC (MD 18.75% IQR 12.2–26.2 vs. MD 17.65% IQR 14.7–23.1, *p* = 0.5177). No significant differences were observed in absolute counts for CD19^+^IL-10^+^ cells between PAC patients and HC (MD (IQR) 131 (62–197), vs. 128 (79–141), respectively, *p* = 0.3214).

**Figure 1:**
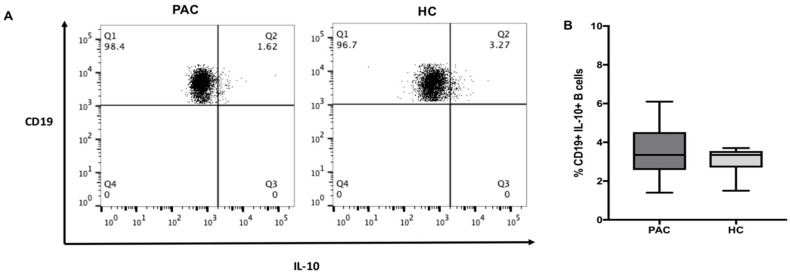
The percentage of CD19^+^IL-10^+^ cells. B cells were identified as the CD19^+^ population, as explained in Figure S1D. (**A**) Representative dot plots of CD19^+^IL-10^+^ cells in PAC and HC. Comparison of the frequency of B cells positive to IL-10 staining (**B**) in both groups. Data are expressed as the mean ± standard deviation (SD).

To identify the percentage of Bregs cells, we determined the percentage of double positive cells to IL-10 and CD19 staining, but we did not find significant differences between groups (PAC = MD 3.35% IQR 1.4–4.5 vs. HC = M 3.35% IQR 1.5–3.5, *p* = 0.6744) (Figure 1). The mean fluorescence intensity (MFI) for IL-10 was evaluated in both groups, and we did not find any significant difference between PAC and HC (MFI MD (IQR) 9207 (5818–19,126), vs. MFI 14460 (12,392–23,392), respectively, *p* = 0.2193).

### 2.2. Diminished Expression of IL-10 in CD19^+^CD38^+^ B Cells in Patients with Perennial Allergic Conjunctivitis

**Figure 2:**
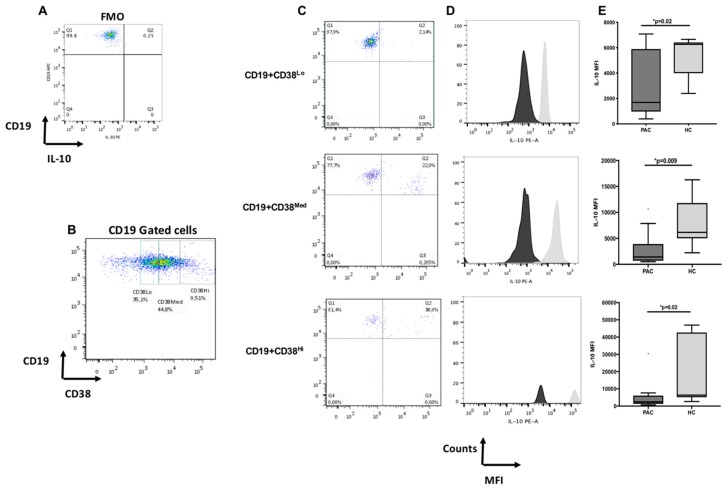
Expression of IL-10 in CD19^+^CD38 cell subsets in patients with perennial allergic conjunctivitis (*PAC*) and healthy controls (HC). Representative dot plots from gates performed according to the CD38 expression on B cells (see Figure S1 to consult the hierarchical analysis performed). Fluorescence minus one (FMO) (**A**); CD38 Lo/Med/Hi on gated CD19^+^ cells (**B**). Representative dot plots of CD19^+^ CD38 Lo/Med/Hi vs. IL-10 staining (**C**); median fluorescence expression (MFI) of IL-10 on gated CD19^+^CD38^+^Lo/Med/Hi cells (**D**). The dark-grey histogram corresponds to PAC patients; the light-grey histogram corresponds to healthy controls; (**E**) column shows the IL-10 MFI comparison between the two groups. See Figure S2 for cytometric control of the IL-10 analysis. Data are expressed as the mean ± standard deviation (SD). Statistical differences were considered when *p* < 0.05. The black dot above PAC columns corresponded to the outlier values.

In addition to determining the percentage of CD19^+^IL-10^+^ B cells, we included identification of the CD38 ectoenzyme on CD19^+^IL-10^+^ B cells, since the density of CD38 expression catalogs three cell subpopulations (CD38Lo, CD38Med, and CD38Hi) on B cells. We did not find differences in CD19^+^CD38^Lo^, CD19^+^CD38^Med^, and CD19^+^CD38^Hi^, nor in CD19^+^CD38^Lo/Med/Hi^ IL-10^+^ cells, between PAC patients and healthy controls. The results are depicted in Table 2. Interestingly, when we analyzed MFI for IL-10 on CD19^+^CD38^Lo/Med/Hi^-gated cells, we observed a significant decrease of MFI in all B cell subsets in patients when compared with HC. Values of MFI were as follows: CD19^+^CD38^Lo^ IL-10^+^ 2866 (PAC) vs. 5406 (HC), *p* = 0.026; CD19^+^CD38^Med^ IL-10^+^ 1417 (PAC) vs. 6153 (HC), *p* = 0.0092; CD19^+^CD38^Hi^ IL-10^+^ 2462 (PAC) vs. 6357 (HC), *p* = 0.0245 (Figure 2).

### 2.3. Increased Concentration of TNF-α and IL-10 in the Tears of Patients with Perennial Allergic Conjunctivitis

**Figure 3:**
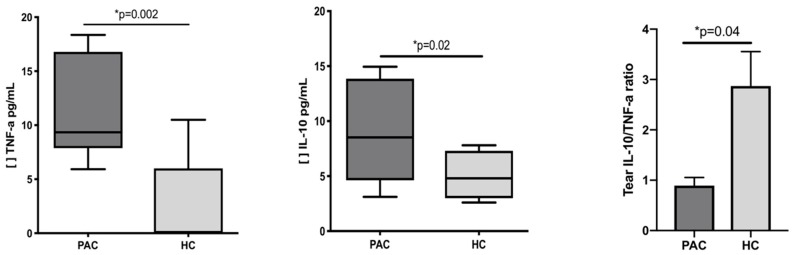
Tear cytokines in patients with perennial allergic conjunctivitis (PAC) and healthy controls (HC). The cytokines IL-10 and TNF were measured by cytometric bead arrays in tear samples from patients with PAC and HC. Significant differences were observed in IL-10 and TNF-α in PAC patients. Analysis of the IL-10/TNF-α ratio showed 2.8 more times tear IL-10 than tear TNF-α in HC. The ratio was calculated using the mean values for IL-10 and TNF-α. See Table 3 for additional determined cytokines. Statistical differences were considered when *p* < 0.05. [ ] Concentration of cytokine.

Tear cytokines were determined in both groups, and the results are presented in Table 3. We observed 2.4 times more tear TNF-α (*p* = 0.002) and 1.9 times more tear IL-10 in PAC patients when compared with healthy controls (*p* = 0.02). Interestingly, when we analyzed the ratio of anti-inflammatory tear IL-10 vs. pro-inflammatory tear TNF-α, we observed 2.8 more times IL-10 than TNF-α in healthy controls when compared with PAC patients (Figure 3). No significant correlations were found between tear cytokines and CD19^+^IL-10^+^ cells, with CD38Lo/Med/Hi IL-10^+^ cell subsets, or with IL-10 MFI.

### 2.4. Functional Evaluation of B Cells after Mitogen Stimulation

**Figure 4:**
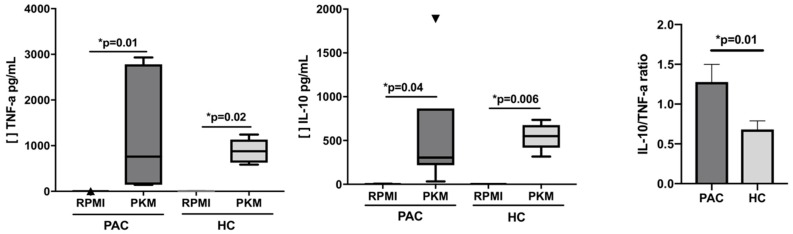
Determination of TNF-α and IL-10 secretion after B cell-stimulation. B cells were stimulated for 24 h with Pokeweed mitogen (PKM). After stimulation, supernatants were collected and cytokines measured by cytometric bead arrays. Significant differences were observed in IL-10 and TNF-α in both groups. Analysis of the IL-10/TNF-α ratio showed 1.2 times more TNF-α than IL-10 in PAC patients when compared with HC. The ratio was calculated using the mean values for IL-10 and TNF-α. See Table 4 for additional determined cytokines. Statistical differences were considered when *p* < 0.05. 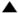 and 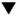 Corresponded to the outliers values. [ ] Concentration of cytokine.

In order to evaluate the secretion of cytokines by B cells, mononuclear cells were stimulated with Pokeweed mitogen (PKM), a known B cell mitogen, over 24 h [17]. No significant differences were observed between PAC patients and HC after stimulation; when the ratio of anti-inflammatory IL-10 vs. pro-inflammatory TNF-α was analyzed in the supernatant of cultured cells, we observed 1.2 times more TNF-α than IL-10 in PAC patients when compared with HC (*p* = 0.01) (Figure 4). The other cytokines evaluated after PKM stimulation are depicted in Table 4.

## 3. Discussion

Allergic conjunctivitis is an inflammation of the conjunctiva secondary to contact with an allergen at the conjunctiva in a previously sensitized individual [18]. Two forms of AC have been described: VKC and AKC are the chronic forms, and can lead to permanent visual impairment, while SAC and PAC are the acute forms, and are the most frequent clinical presentations. Immunologically, CD4 T cells have been described as the main cell subsets that control conjunctival inflammation in both the acute and chronic forms [5,6,19], but a diminished frequency of Tregs has been reported in PAC patients [8]. This is remarkable due to the growing evidence that suggests that the induction of Tregs is maintained by a subset of IL-10-expressing B cells (Bregs) [11,12,13]. Underlining the importance of Bregs in allergic conjunctivitis, Miyazaki et al. demonstrated in a mice model of IL-10 deficient B cells an exacerbated late-phase inflammation, whereas transfer of IL-10 secreting B cells protected against inflammation [20], suggesting a regulatory function for B cells in allergic conjunctivitis. Nonetheless, the evaluation of Bregs in patients with PAC had not been described yet.

In this work, we analyzed the frequency of Bregs and the expression of IL-10 in Bregs subsets in patients with PAC. Our results are in accordance with those of other authors that have reported changes in the frequency of B cell subsets involved in allergic inflammation, including CD27^+^CD24^hi^, CD24^hi^CD38^hi^ and CD73^−^CD25^+^CD71^+^ cells [15]. Remarkably, these Bregs populations have been observed after allergen-specific desensitization, or in nonallergic individuals highly exposed to allergens [15]. In our work, we did not find differences in the percentage or absolute numbers of CD19^+^IL-10^+^ cells between patients and controls, but analysis of IL-10 MFI in circulating CD19^+^CD38^Lo/Med/Hi^ B cell subsets showed they were markedly diminished in PAC patients. The MFI result is translated as low expression of IL-10 in CD19^+^CD38^+^ B cells [21]. The absolute numbers and percentage of CD19^+^ cells observed in our groups are in accordance with other authors [22,23,24] who have reported normal variations in the reference values for B cell subpopulations from infancy to adulthood. Those reports recognized that B cells decline with age, showing greater percentages at birth and infancy, declining gradually during childhood, and after adolescence, B cells reach values similar to those found in adults [22,23,24]. Also, CD38 has similar expression kinetics with higher values at infancy, declining with age [23,24]. Iwata et al. reported a “rare” IL-10 B cell subpopulation in blood from adult donors, similar to the spontaneous IL-10 producing B cells in mice, that corresponded to the 0.6% of total B cells [25]. Thus, it is possible that the percentage of IL-10^+^ cells in the circulating CD19^+^CD38^+^ cells observed in our study would be the biological reflex of a normal variation during childhood. Blair et al. have suggested that CD24 and CD38 define a subset of B cells with regulatory characteristics [26], and in mice models, it has been recognized that CD38 is a protein that enhances IL-10 production by regulatory B cells [27]. Circulating phenotypes of IL-10^+^ B cells in both human and mice models have been described, and changes in the frequency of circulating IL-10^+^ B cells are associated with dysregulation of immune responses [28,29]. Moreover, it has been reported that human CD19^+^CD24^hi^CD38^hi^ B cells exhibit regulatory capacity in healthy individuals, while the same B cells produce less IL-10 and lack suppressive activity in SLE patients. In line with the findings of these authors, when we analyzed IL-10/TNF-α production in B stimulated cells, we observed an inverted IL-10/TNF-α ratio in PAC patients when compared to healthy controls. These differences between groups indirectly indicate saccharidic changes in the cells of patients with PAC, since polyclonal activation of PKM occurs in their lectin binding to (GlcNAc)3 structures that are mainly expressed on B cells, but also on some subsets of T and NK cells [30]. Although saccharidic specificity is essential for cell activation, proliferating activity and immunoglobulin production depend on the interaction with TLRs, mainly TLR2 and TLR9 [17]. Glycophenotype evaluation could give new insights into the ocular allergy pathophysiology, since cytokine production is a direct consequence of cell activation induced by PKM.

In line with this, changes in cytokines also induce glycosylation changes in cells, regulating cell-cell interaction and inflammation [31]. Unfortunately, in this study, neither proliferative activity nor induced immunoglobulin production by PKM stimulated cells were evaluated. The lack of a complete B cell evaluation in this work is a limitation to being able to conclude that the differences observed are associated with differential expression of TLRs in PKM stimulated cells, and further assessment is needed. It is important to note that stimulation assays were performed with peripheral blood monononuclear cells (PBMC) thus, the cytokines reported in this work were produced not only by B cells, but also other cells such as T cells and macrophages also contributed to the observed result. Regardless, this does not change the biological fact that PAC patients responded immunologically differently compared with HC during PKM stimuli, however, additional assays are needed to find whether the circulating CD19^+^CD38^+^IL-10^+^ cells from PAC patients are dysfunctional.

IL-10 is a cytokine that is described as a potent anti-inflammatory molecule with paradoxical functions [32]. Its anti-inflammatory functions include the downregulation of T cells and monocyte activation, inhibiting Th1 mediated inflammation. Nonetheless, IL-10 does not impair the ability of B cells to function as antigen presenting cells. Moreover, IL-10 contributes to the stimulation of Th2 cytokines [33]. In line with its Th2-promoting functions, IL-10 has been recognized as a potent stimulatory factor for mast cells and their progenitors [34]. The activation of mast cells in a Th2 microenvironment is a critical characteristic in allergic conjunctivitis inflammation [35]. At the ocular surface level, IL-10 is produced by conjunctival fibroblasts [36], by blood inflammatory cells such as basophils and eosinophils, and by local mast cells after an allergenic challenge [37,38]. Some authors have suggested a pathogenic role of IL-10 in ocular surface inflammatory diseases in which the Th2 profile is associated with ocular damage [39,40], and in a mice model of induced allergic conjunctivitis, IL-10 contributed to the development of the acute effector phase, augmenting the infiltration of eosinophils into the conjunctiva [41]. These paradoxical functions of IL-10 reinforce our observation of increasing tear IL-10 in patients when compared with control subjects, and at the same time, an inverted IL-10/TNF-α ratio, approximating to the Th2/Th9 mediated inflammation reported in experimental conjunctivitis [42]. TNF is one of the major mediators of inflammation and is a pleiotropic cytokine produced by diverse cell types such as macrophages, NK cells, T cells, fibroblasts, and mast cells [43]. Conjunctival epithelial cells stimulated with TNF-α induce the secretion of MCP, IL-1β, and IL-6 [44], all of which are molecules involved in the pathogenesis of human ocular allergy [45]. Remarkably, stimulation of corneal and conjunctival fibroblasts with TNF- α induces eosinophil chemotaxis [46], and at the ocular level, the resident mast cells are the primary source of TNF-α in the allergic process [47], sustaining the ocular allergic inflammation. On the contrary, increased IL-10 has been reported in the contralateral healthy eye when infection is occurring in the other eye, supporting the idea that IL-10 has an anti-inflammatory function [48]. Whether tear IL-10 is a participant in the local Th2 inflammation or is an attempt to inhibit the pro-inflammatory actions of TNF-α at the ocular surface is not known, and further investigations are needed to clearly define the role of tear IL-10 in human allergic conjunctivitis, as well as the involvement of IL-10 activated mast cells.

Understanding systemic and local mechanisms involved in allergic conjunctivitis will aid in designing immunomodulating therapies at different levels. Our results ratify the notion of the medical ophthalmological use of specific immune therapy (SIT) for acute types of ocular allergy (SAC and PAC), as it has been reported for other allergies. In asthma, SIT with allergens induces IL-10 producing B cells [15]. The induced Bregs suppress the allergen-specific immune response, contributing to tolerance [49,50]. Luo et al. reported changes in B cell subsets in allergic rhinitis, associating an increased percentage of terminally differentiated CD19^+^CD24^hi^CD38^+^cells, a subset of Bregs, with better clinical outcomes in patients receiving immunotherapy [51]. On the other hand, at the ocular level, the addition of IL-10 could have paradoxical results. For example, in a mouse model of uveitis, distinct doses of IL-10 showed contradictory effects, and low doses of IL-10 potentiated LPS induced inflammation, while high IL-10 doses induced anti-inflammation [52]. In contrast, using adenovirus vectors encoding for IL-10 in human conjunctival cells [53] and lacrimal gland epithelial cells [54] has demonstrated control of lymphocyte proliferation. Thus, IL-10 seems to play a dual role in the ocular microenvironment by simultaneously suppressing proinflammatory pathways and enhancing others, depending on the dose if IL-10 is administered intraocularly or induced at the surface. These opposite roles must be taken into account during the development of biological therapies using IL-10 at the ocular level.

**Figure 5:**
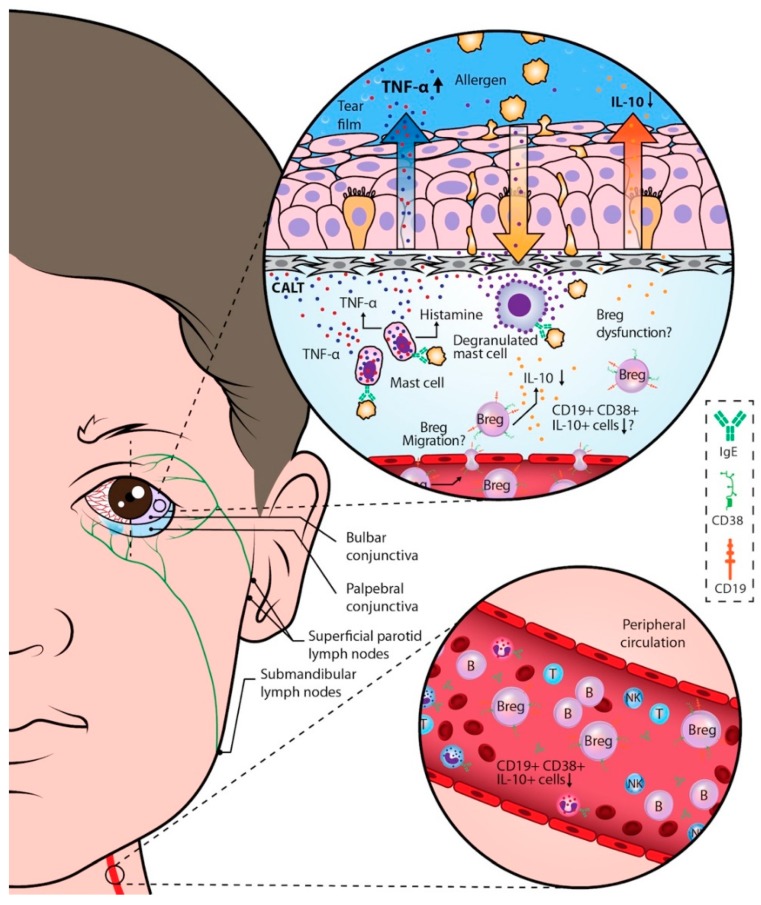
Systemic and local immune dysregulation observed in patients with perennial allergic conjunctivitis (PAC). The immune dysregulation in PAC is characterized by the low expression of IL-10 in circulating CD19^+^CD38^+^ B cells (Bregs), favoring a systemic Th2 response that is clinically observable with higher concentrations of IgE in the serum of patients with PAC. Whether these IL-10^+^ B cells are migrating to the conjunctiva or are dysfunctional B cells unable to down-regulate the Th2 immune response is unknown, and needs further investigation. IL-10 predominates over TNF-α in tears under physiological conditions of the ocular surface; nevertheless, in PAC patients, an inverted IL-10/TNF-α ratio is observed. Tear TNF-α facilitates the migration of circulating blood cells to the conjunctiva, and at the same time, the activation of local sensitized mast cells by allergens contributes to more inflammation through the secretion of TNF-α. Amplification of the ocular damage is observed each time that the allergens cross the conjunctival epithelium, activating the sensitized local o migratory cells. For example, eosinophils are one such cell of this type which injure the cornea and other structures of the ocular surface. Thus, a damaging circle is created in which the tear cytokines IL-10 and TNF-α appear to be the biomolecules directing the ocular allergic process; this circle is reinforced with the systemic feedback of possible dysfunctional circulating Bregs. CALT: Conjunctival Associated Lymphoid Tissue; B: B cells; T: T cells; NK: Natural Killer cells.

Our findings demonstrate an immunological dysregulation in patients with allergic conjunctivitis, characterized by the low expression of IL-10 in circulating CD19^+^CD38^+^ Bregs subsets, and an inverted tear IL-10/TNF-α ratio (Figure 5). Ophthalmological therapeutics should consider systemic and local interventions to reduce ocular damage in patients with allergic conjunctivitis.

## 4. Materials and Methods

### 4.1. Patients and Health Controls

Sixteen patients with perennial allergic conjunctivitis (PAC) (11 males and 5 females, mean age 12.1 years, range 6–15) were included in the study. PAC diagnosis was based on clinical ophthalmological history (mean disease duration 3.5 (SD 3.1) years) and allergo/immunological examination. All patients were classified as having active forms of PAC. The allergic condition was confirmed with a skin-prick test positive for *Der p* (wheal ≥ 3 mm diameter, and compared with histamine control) and determination of serum total IgE (tIgE) and specific IgE (sIgE) to *Dermatophagoides pteronyssinus* 1 (*Der p* 1).

Eight healthy volunteers were used as controls (4 males and 4 females, mean age 12.5 years, range 7–17). All participants in this study gave their assent consent for blood sampling after written information was provided. The study adhered to the ethical principles of the Declaration of Helsinki and the E11 Statements of the International Conference of Harmonisation (E11-ICH). This work was approved by the Scientific (CI-001-2016, 08/February/2016), Bioethics (CEI-2016/01/01, 09/March/2016), and Biosafety (CB-001-2016, 29/March/2016) Institutional Committees at the Institute of Ophthalmology, “Foundation Conde de Valenciana”, Mexico City.

### 4.2. Monoclonal Antibodies and Reagents

Allophycocyanin (APC)-labeled-mouse monoclonal antibodies (mAbs) against human CD19, and phycoerythrin (PE)-labeled mAbs anti-human IL-10, were purchased from BioLegend (San Diego, CA, USA). PE-Cy7-labeled mAbs anti-human CD38 were acquired from BD Biosciences (San Jose, CA, USA), and BD™ CompBeads (San Diego, CA, USA). BD FACS™ Lysing Solution, BD Cytofix/Cytoperm™ solution, and BD FACSFlow™ Sheat Fluid were purchased from BD Biosciences (San Jose, CA, USA). RPMI-1640 culture medium, Pokeweed mitogen (PKW), and salts were purchased from Sigma Chemical Co. (St. Louis, MO, USA). Sodium pyruvate, L-glutamine, and 2-mercaptoethanol were purchased from Gibco BRL (Rockville, MD, USA). Fetal calf serum was obtained from HyClone Labs (Logan, UT, USA), and *Dermatophagoides pteronyssinus* (*Der p*) was purchased from Allerstand Co. (Mexico City, Mexico).

### 4.3. Peripheral Blood Samples

Blood samples were collected by venipuncture into a BD Vacutainer™ K2 EDTA tube (BD, Franklin Lakes, NJ, USA). The sample was kept in gentle agitation and at room temperature (25 °C) for ~30 min for subsequent immunofluorescence staining of the cell surface and intracellular markers.

### 4.4. Immunofluorescence Staining of Cell Surface Markers

Two-color staining was performed on the peripheral blood cells with direct immunofluorescence, using either APC-mAb anti-CD19 or PE-Cy7-mAb anti-CD38. Briefly, 20 µL of whole peripheral blood was incubated with fluorochrome-labeled mAb for 30 min at 25 °C in darkness. After incubation, the red blood cells were lysated with BD FACS™ Lysing Solution according to the manufacturer’s instructions; then, the cells were washed twice with BD FACSFlow™ Sheat Fluid and processed with intracellular staining.

### 4.5. Immunofluorescence Staining of Intracellular Markers

After extracellular staining was performed, the cells were fixed and permeabilized with BD Cytofix/Cytoperm™ solution according to the manufacturer´s instructions. The cells were then incubated with PE-labelled anti-human IL-10 antibody, and immediately acquired by flow cytometry. In all cases, Fluorescence Minus One (FMO) controls and anti-mouse Ig and κ/Negative control compensation particle sets (BD™ CompBeads) were used. All samples were immediately acquired after immunoflurescence staining.

### 4.6. Flow Cytometric Analysis

All cells were analyzed for the expression of phenotypic markers on a BD FACSVerse™ (BD Biosciences, San Jose, CA, USA) flow cytometer using FACSuite Software version 1.0.5.3841 (BD Biosciences, San Jose, CA, USA), and 10,000 events were counted. To analyze the staining of the cell-surface markers, single cells were first gated (FSC-H-forward height vs. FSC-A-forward area); subsequently, a second gate was drawn according to the physical properties (FSC-forward and SSC-scatter) that corresponded to lymphocytes. Then, CD19^+^ cells were selected in a third FSC-CD19 dot. To determine the subsets of Bregs cells, a new dot plot was obtained showing CD19^+^ cells (y-axis) and CD38^+^ cells (x-axis). Subsequently, we identified CD38 expression as low, medium, and high sub-populations on CD19^+^ B cells (see Figure S1). Finally, using intracellular IL-10 staining on gated CD19^+^ and CD19^+^CD38^Lo/Med/Hi^ B cells, a dot plot and/or a histogram was drawn to analyze the frequency or the mean fluorescence intensity (MFI) of IL-10^+^ cells. Control staining was performed using fluorescence minus one (FMO) and BD™ CompBeads.

### 4.7. Tear Samples

Tear samples from healthy and allergic eyes were obtained from the conjunctival fornix by addition of 20 µL of BSS™ sterile saline solution (Alcon Laboratories, Inc., Fort Worth, Texas, USA) at the ocular surface, according to Santacruz´method [55]. Then, the ocular wash was immediately recovered and stored at −20 °C until cytometric analysis.

### 4.8. Serum Samples

A sample of peripheral blood was taken into a BD Vacutainer™ serum tube (BD, Franklin Lakes, NJ, USA), allowing the blood to clot for ~20 min. Then, the serum was obtained by centrifugation at 130 *g* × 10 min and stored at −20 °C until tIgE and sIgE determination by fluoroenzymo immuno assay with the ImmunoCAP Phadia^®^ 100 Laboratory System (Thermo Fisher Scientific Inc., Portage, MI, USA).

### 4.9. Cell Cultures

PBMC were cultured in 24-well flat bottomed cell culture plates (Costar, Cambridge, MA, USA) at 5 × 10^5^ cells/well in Roswell Park Memorial Institute (RPMI) 1640 (RPMI-1640) medium, supplemented with 1 mM sodium pyruvate, 2 mM L-glutamine, and 0.5% heat-inactivated fetal calf serum, and incubated at 37 °C in a 5% CO2 humidified chamber. After 24 h the culture medium was removed, and fresh culture medium was supplemented with 10% heat-inactivated fetal calf serum and Pokeweed mitogen (PKW) (5 µg/mL). After 24 h, the supernatants were collected and stored at −70 °C to measure the soluble cytokines.

### 4.10. Determination of Tear and Serum Cytokines

IL-8, IL-1β, IL-6, IL-10, TNF-α, and IL-12p70 (Human Inflammatory Cytokine Kit, BD Biosciences, San Jose, CA, USA) were measured with Cytometric Bead Arrays (CBA) in tear and serum samples, according to the manufacturer´s instructions (BD Biosciences, San Jose, CA, USA). The samples were processed by flow cytometry, and the results were analyzed with FCAP Array™ Software version 3.0 (BD Biosciences, San Jose, CA, USA).

### 4.11. Determination of Total IgE (tIgE) and Specific IgE (sIgE)

The serum samples were processed to determine tIgE and sIgE to *Der p* 1 by fluorenzymeimmunoassay (FEIA), following the manufacturer’s instructions, in an ImmunoCAP Phadia^®^ 100 Laboratory System. The results were analyzed by ImmunoCAP^®^ v.4.13 software (Thermo Fisher Scientific Inc., Portage, MI, USA), and the limits of detection were <2 kUI/L for Total IgE, and <0.1 kUA/L for sIgE.

### 4.12. Statistical Analysis

A Mann–Whitney U test was used to determine significant differences; correlations between the studied variables were performed with the Spearman rank test; and *p* < 0.05 was considered as statistically significant. The analysis was performed using GraphPad Prism software version 7.00 for Windows (GraphPad Software, La Jolla, CA, USA).

## 5. Conclusions

Our findings demonstrate an immunological dysregulation in patients with allergic conjunctivitis, characterized by the low expression of IL-10 in circulating CD19^+^CD38^+^ Bregs subsets, and an inverted tear IL-10/TNF-α ratio. These findings highlight the novel pathologic changes involved in ocular allergic diseases.

## Figures and Tables

**Table 1:** Demographics characteristics of patients with PAC and healthy controls.

Demographic Characteristics	PAC	HC	*p* Value
	(*n* = 16) MD (IQR)	(*n* = 8) MD (IQR)	
Age	12.5 (11–14)	12 (11–15.5)	NS
Male	13 (11–14)	11 (11–13.3)	NS
Female	11 (10.5–13.5)	14.5 (8.5–16.8)	NS
TBUT (sg)	4.5 (3–6)	6 (3.5–8)	NS
Schirmer Test (mm)	16.5 (9–27)	20 (14.8–29.8)	NS
SPT (mm) to *Der p*	6 (3–8)	0 (0–0)	0.0006
IgA	174 (140–211)	171 (86–213)	NS
IgG	1230 (1169–1377)	1310 (649–1933)	NS
IgM	116 (85–146)	127 (103–516)	NS
tIgE	404 (164–585)	58.3 (29.3–182.5)	0.002
sIgE	29.9 (11.4–62.9)	0 (0–0) *	<0.0001

PAC: Perennial Allergic Conjunctivitis; HC: Healthy Controls; MD: Median; IQR: Interquartile Range; NS: Not Significant; TBUT: Tear Breakup Tear; SPT: Skin Prick Test; *Der p: Dermatophagoides pteronyssinus*; tIgE: Total IgE; sIgE: Specific IgE. * Results below the limit of detection were considered as 0 for statistical analysis. See Table S2 for a detailed description of demographic characteristics.

**Table 2:** Percentages of B cell subsets in patients with perennial allergic conjunctivitis (PAC) and healthy controls (HC).

B Cell Subsets	PAC	HC	*p* Value
	(*n* = 16)	(*n* = 8)	
	MD (IQR)	MD (IQR)	
CD19^+^	18.8 (16.9–21.9)	17.7 (15.7–20.1)	0.5177
CD19^+^CD38^+^	84.4 (77.9–88.6)	86 (71.9–91.8)	0.9649
CD19^+^CD38^Lo^	61.5 (36.8–64.1)	55.2 (40.6–60.5)	0.2597
CD19^+^CD38^Med^	23.5 (14.2–31.3)	17.5 (7.2–36.7)	0.6650
CD19^+^CD38^Hi^	5 (3.0–11.9)	5.25 (1.8–14.9)	0.2834
CD19^+^IL-10^+^	3.8 (2.7–4.6)	3.3 (1.9–3.5)	0.2198
CD19^+^CD38^Lo^ IL-10^+^	2.5 (1.6–4.2)	2.3 (1.0–7.0)	0.500
CD19^+^CD38^Med^ IL-10^+^	5 (2.6–6.7)	4.8 (3.7–5.4)	0.449
CD19^+^CD38^Hi^ IL-10^+^	4.7 (3.2–7.3)	10.4 (3.9–15.3)	0.419

PAC: Perennial Allergic Conjunctivitis; HC: Healthy Controls; MD: Median; IQR: Interquartile Range.

**Table 3:** Tear cytokines in patients with perennial allergic conjunctivitis (PAC) and healthy controls (HC).

Tear Cytokines	PAC (*n* = 16)	HC (*n* = 8)	*p* Value
	MD (IQR)	MD (IQR)	
	pg/mL	pg/mL	
IL-1β	32.46 (19.85–47.63)	33.8 (18.02–38.79)	0.4435
IL-6	25.46 (14.61–40.41)	19.95 (17.36–25.28)	0.3333
IL-8	167 (127–222.7)	176 (158–245)	0.3031
IL-10	8.5 (4.6–13.9)	4.8 (3.0–7.3)	0.02
IL-12p70	27.17 (7.9–39.53)	18.6 (7.9–22.6)	0.1750
TNF-α	9.34 (7.88–16.79)	0 (0–6.0) *	0.002

Kit detection limits were as follows: IL-8, 3.8 pg/mL; IL-1β, 7.2 pg/mL; IL-6, 2.5 pg/mL; IL-10, 3.3 pg/mL; TNF-α, 3.7 pg/mL; and IL-12p70, 1.9 pg/mL. * Results below the limit of detection were considered as 0 for comparison between groups.

**Table 4:** Supernatant (SN) cytokines after Pokeweed mitogen (PKM) stimuli in cells from patients with perennial allergic conjunctivitis (PAC) and healthy controls (HC).

SN Cytokines	PAC (*n* = 16)			HC (*n* = 8)		
	MD (IQR)			MD (IQR)		
	pg/mL			pg/mL		
	RPMI	PKM	*p*	RPMI	PKM	*p*
IL-1β	6.6 (5.2–7.6)	454.7 (108.8–1342)	0.03	6.3 (5.9–6.9)	830 (380–1161)	0.01
IL-6	3.8 (3.1–4.6)	3078 (2137–8638)	0.03	4.8 (4–9.8)	5084 (2508–6197)	0.002
IL-8	919 (318–1043)	20,237 (10,558–21,513)	0.0003	1223 (501.5–1654)	22,075 (17,873–24,910)	0.004
IL-10	0 (0–0.3) *	304.8 (218–866)	0.006	3.3 (0–4.6) *	550.2 (418–675)	0.04
IL-12p70	4.6 (2.2–4.9)	21 (4.5–50)	0.02	3.5 (2.2–4.5)	8.9 (4.8–47.3)	0.03
TNF-α	0 (0–1.9) *	877 (627–1131)	0.01	0 (0–1.2) *	757.6 (144.9–2932)	0.02

Kit detection limits were as follows: IL-8, 3.8 pg/mL; IL-1β, 7.2 pg/mL; IL-6, 2.5 pg/mL; IL-10, 3.3 pg/mL; TNF-α, 3.7 pg/mL; and IL-12p70, 1.9 pg/mL. * Results below the limit of detection were considered as 0 for comparision between groups.

## Supplementary Materials

The supplementary materials can be found at https://www.mdpi.com/1422-0067/20/5/1035/s1.
