# Supplementary material for: Low Expression of IL-10 in Circulating Bregs and Inverted IL-10/TNF-α Ratio in Tears of Patients with Perennial Allergic Conjunctivitis: A Preliminary Study
Source: Int J Mol Sci. 2019 Feb 27;20(5):1035. doi: 10.3390/ijms20051035 (PMC6429471; doi:10.3390/ijms20051035)
Supplement: Supplementary file 1 [file ijms-20-01035-s001.zip › ijms-431425 sp for final/Supplementary Figure Legends.pdf]

Supplementary Figure S1. Identification of IL-10+ B Cells, and B Cell Subsets by Flow Cytometry. First, Singlets events were selected (A), then CD19+ cells were gated within the lymphocyte gate (B-C), next a dot plot was created to show CD19 vs IL-10 or CD19 vs CD38 staining (D-E).

Supplementary Figure S2. IL-10 analysis in CD19+ Enriched Cells. CD19 + cells from a healthy subject were enriched by negative selection (RossetteSep™, STEMCELL Technologies, Vancouver, BC, Canada) according to the manufacturer's specifications, (Upper histograms). Then, enriched cells were cultured in RPMI 1640 medium free of fetal bovine serum (FBS). After 8 h of culture, RPMI medium was removed, and fresh supplemented culture medium with L-glutamine (Gibco BRL, Rockville, MD, USA), and 10% of FCS (HyClone Labs, Logan, UT, USA) was added. Then, the enriched cells were stimulated for 24h with 5 or 10 μg of LPS from E. coli OIII: B4 (Sigma Chemical Co. St. Louis, MO, USA). Five 5h before culture ended, 1μg/mL of Brefeldin A was added; after that, cultured B cells were harvested, fixed, and permeabilised (Fixation/Permeabilisation Solution Kit with BD GolgiStop™) for anti-IL-10PE staining. Cells were immediately acquired and analysed by flow cytometry. Dot plots show a clearly defined IL-10+ cell subset inside B isolated cells.
