# Supplementary figures and images for: Low Expression of IL-10 in Circulating Bregs and Inverted IL-10/TNF-α Ratio in Tears of Patients with Perennial Allergic Conjunctivitis: A Preliminary Study
Source: Int J Mol Sci. 2019 Feb 27;20(5):1035. doi: 10.3390/ijms20051035 (PMC6429471; doi:10.3390/ijms20051035)

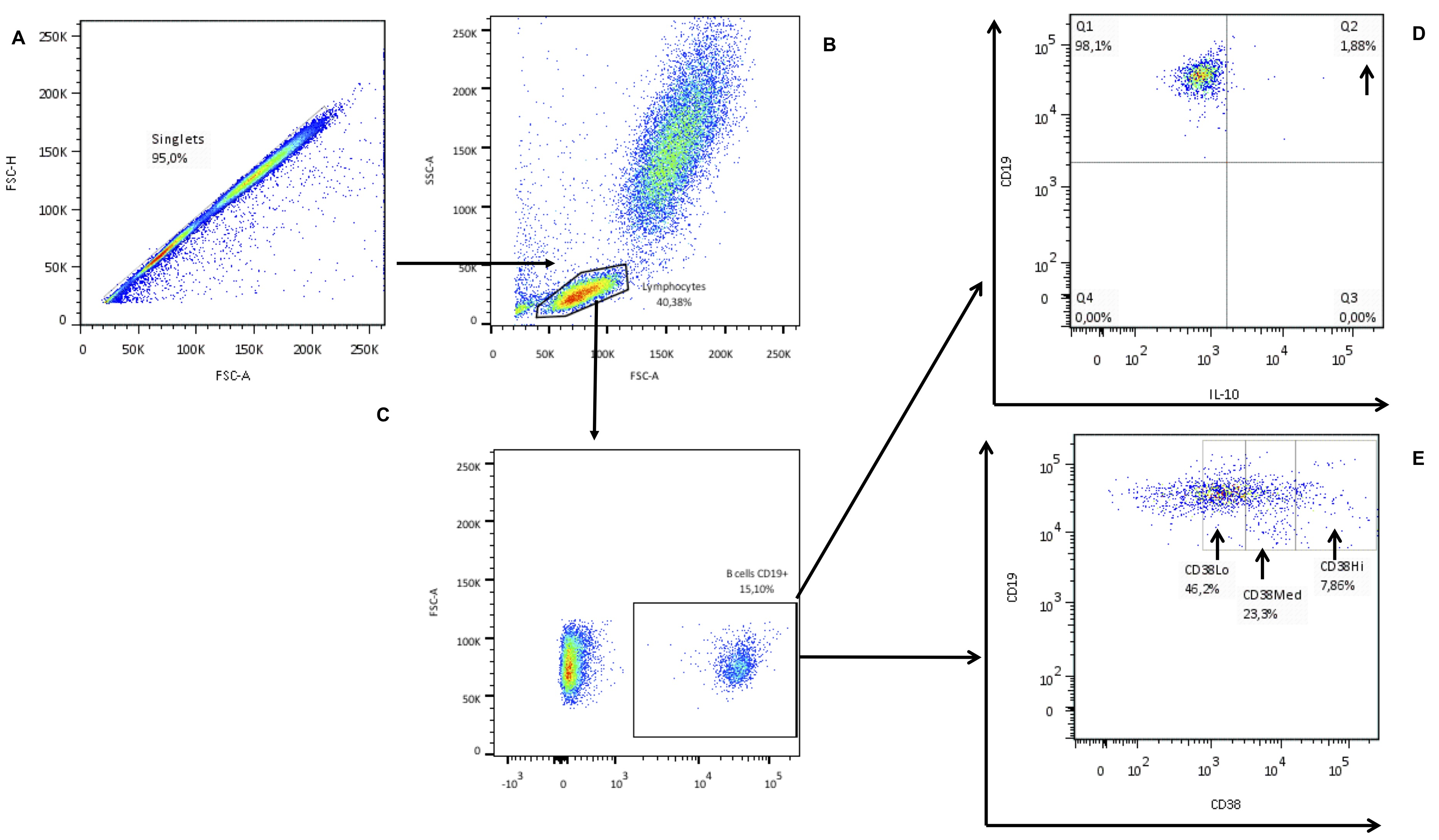

Supplement: Supplementary file 1 [file ijms-20-01035-s001.zip › ijms-431425 sp for final/Figure S1.tiff]

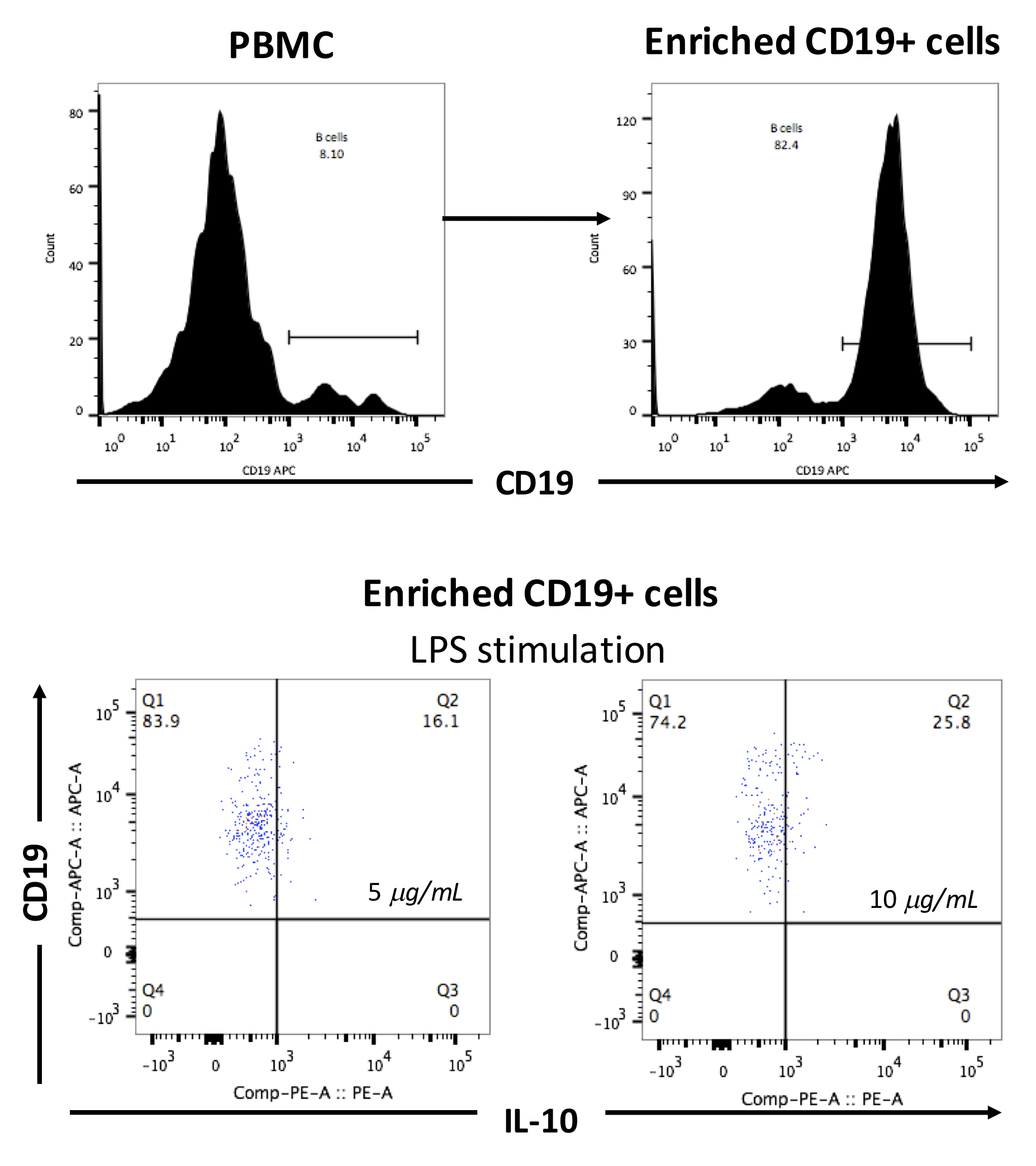

Supplement: Supplementary file 1 [file ijms-20-01035-s001.zip › ijms-431425 sp for final/Figure S2.tiff]
